# Phylogenetic assessment of filoviruses: how many lineages of Marburg virus?

**DOI:** 10.1002/ece3.297

**Published:** 2012-07-01

**Authors:** A Townsend Peterson, Mark T Holder

**Affiliations:** Department of Ecology and Evolutionary Biology, The University of KansasLawrence, Kansas, 66045

**Keywords:** Ebola virus, filovirus, lineage identity, Marburg virus

## Abstract

Filoviruses have to date been considered as consisting of one diverse genus (Ebola viruses) and one undifferentiated genus (Marburg virus). We reconsider this idea by means of detailed phylogenetic analyses of sequence data available for the *Filoviridae*: using coalescent simulations, we ascertain that two Marburg isolates (termed the “RAVN” strain) represent a quite-distinct lineage that should be considered in studies of biogeography and host associations, and may merit recognition at the level of species. In contrast, filovirus isolates recently obtained from bat tissues are not distinct from previously known strains, and should be considered as drawn from the same population. Implications for understanding the transmission geography and host associations of these viruses are discussed.

## Introduction

Filoviruses have been known to science for four decades, and have been the focus of many detailed studies and analyses. In particular, Marburg virus was first noted in 1967, in outbreaks in Europe imported via laboratory primates from Uganda (Monath 1999); subsequent hemorrhagic fever outbreaks placed it in Kenya (Smith et al. 1982; Johnson et al. 1996), Zimbabwe (Conrad et al. 1978), Democratic Republic of the Congo (DRC; Bausch et al. 2006), and Angola (Towner et al. 2006). Ebola virus made a two-species debut in 1976, with near-simultaneous hemorrhagic fever outbreaks in Sudan (Anonymous 1978a) and DRC (Anonymous 1978b). Subsequent appearances have been episodic, with detections in Sudan, DRC, Gabon, Republic of Congo, Ivory Coast, and Uganda (Peterson et al. 2004a; Groseth et al. 2007; Towner et al. 2008), as well as importations of the Ebola Reston species into the United States and elsewhere (Rollin et al. 1999).

The current taxonomy of filoviruses recognizes five species: a single Marburg virus species, and four Ebola virus species (Peters et al. 1993; Netesov et al. 2002; Jahrling et al. 2003). The Ebola species are all apparently allopatric or parapatric with respect to one another: Ebola Ivory Coast isolated in West Africa, Ebola Sudan in southern Sudan and Uganda, Ebola Zaire in the Congo Basin, and Ebola Reston possibly from the Philippines (Taniguchi et al. 2011). A recent publication (Towner et al. 2008) documents what appears to be a fifth species, from Uganda, and applies to it the name *Bundibugyo ebolavirus*, or Ebola Bundibugyo for the purposes of this paper. The geographic arrangement of Ebola virus species has been interpreted as suggestive of association with a clade of reservoir species or with a single geographically structured reservoir species (Peterson et al. 2004b, 2007), in contrast to the apparently relatively undifferentiated Marburg virus.

The phylogeny and evolutionary history of the filoviruses, nonetheless, remain poorly understood. Previous phylogenetic analyses have either used dubious approaches to phylogeny estimation and interpretation (Suzuki and Gojobori 1997), or have been vague or imprecise in describing methodologies used (Sanchez et al. 1998; Towner et al. 2006; Swanepoel et al. 2007; Towner et al. 2007). Indeed, among the few analyses for which phylogenetic methodologies are described in detail, results have yielded conflicting interpretations of evolutionary history and geographic sequences, as illustrated by recent debates regarding purported wave-like spread of Ebola Zaire across the Congo Basin (Walsh et al. 2005; Wittmann et al. 2007).

The phylogeny of the filovirus clade nonetheless has important implications for understanding the geography and host (reservoir) relationships of these viruses. Herein, we present no new sequence data, but rather a first careful analysis of existing sequence data, paying particular attention to isolates that may represent distinct lineages. We use detailed phylogenetic analyses and simulations based on coalescent theory to assess the relationships and species status of key filovirus lineages: the RAVN Marburg strain (Johnson et al. 1996) and filovirus sequences derived from several bat tissue samples (Leroy et al. 2005; Swanepoel et al. 2007; Towner et al. 2007). Using novel analytical approaches, we assess the degree of phylogenetic cohesion of particular species units, providing a novel view of filovirus evolution that emphasizes two distinct Marburg lineages in Africa.

## Methods

We initially searched GenBank (http://www.ncbi.nlm.nih.gov/) for all nucleotide sequence data sets described as “Ebola,” “Marburg,” or “filovirus.” The resulting sequences were then filtered carefully, removing (1) sequences that have seen extensive laboratory alteration, (2) duplicate or related suites of sequences derived from the same initial virus isolate, or (3) multiple strains derived from the same outbreak when only a single index case was known (which probably represent evolutionary change outside of the long-term reservoir host). (Note that this step makes the tests described below more conservative, as short branch tips would make intraclade distances look shorter, while leaving basal branches unchanged; see below). In the case of duplicate sequences, we chose reference sequences whenever possible, and always chose the most complete sequence available. In all, 65 individual sequences were assembled, of which 14 were complete genome sequences, 11 were from Ebola virus, and 55 were from Marburg virus. As a general summary, the reference reads included coding sequence from the nucleoprotein, VP35, VP40, Glycoprotein, VP30, VP24, and Polymerase (L) genes, whereas the bat sequences and many from the DRC were fragmentary, including only part of VP35 or the VP35 and L (polymerase) genes.

Initial inspections of data indicated dramatic sequence differentiation between filovirus species and genera, which greatly complicates sequence alignment. Indeed, three rather extreme steps proved necessary before we could be at all confident in alignments (cf. Walsh et al. 2005). (1) Ebola and Marburg virus strains were analyzed separately, as differentiation (even in coding regions) was so extreme as to prevent rigorous alignment. Rather, we assumed (reasonably, at the time; see Discussion) that these two morphologic unique virus lineages are monophyletic and sisters, and that they would be connected by a long, basal branch. (2) We eliminated all noncoding regions by comparison with well-documented reference sequences; noncoding regions were simply too variable to permit alignment even of congeneric species (see fig. 4 in Towner et al. 2006). Finally, (3) we identified areas even within coding regions where initial alignments were highly unstable (i.e., where single-base gaps were inserted to line up single bases). This third step removed 11.9% of the coding regions of the filovirus genome from analysis; analyses with and without these unstable regions yielded identical overall tree topologies, but we were considerably more confident in the data set having taken step (3). Alignments for the Ebola sequences were conducted in ClustalX (Thompson et al. 1997), and reviewed and edited by eye in BioEdit version 7.0.9.0 (Hall 1999). The alignment of Marburg sequences contained a total of 55 sequences (many of which were incomplete); these sequences were aligned using MAFFT version 6.240 (Katoh et al. 2002, 2005). The aligned, trimmed data sets are available at http://hdl.handle.net/1808/9875.

For the Ebola virus sequences, we produced Bayesian estimates of the phylogenetic tree from the entire alignment, and as separate estimates for each of the seven genes (GP, L, NP, VP24, VP30, VP35, and VP40). The data were analyzed under the general time reversible model of evolution (Lanave et al. 1984; GTR hereafter), with invariant sites (I) and gamma-distributed (Γ) rate heterogeneity (Yang 1994). In combined data analyses, sites were partitioned by gene; parameters for the models were estimated separately for each subset, including a relative rate parameter for the subset. All of these analyses were conducted in MrBayes version 3.2 (Ronquist and Huelsenbeck 2003) under unconstrained branch-length priors and using four “heated” chains and four independent Markov chain Monte Carlo (MCMC) runs; the automated stopping rule of MrBayes was used to control length of the MCMC simulation. Analytical results were summarized as majority-rule consensus trees. Slightly more structure could be discerned when we assess the maximum a posteriori (MAP) tree or maximum likelihood tree, but none of those “extra” branches was well-supported, so we present the more conservative, well-supported results only.

The focus of our analysis of Marburg virus sequences was on the phylogenetic cohesion of the sequences. In particular, we concentrated on the question of whether splits between sequences of special interest and better-known sequences were unexpectedly deep. For analyses of the RAVN strain, we addressed the question of whether the genetic dissimilarity between the two RAVN sequences available and the remaining Marburg sequences was larger than one would expect, if all of the sequences were to be derived from a single species. Because many sequences were incomplete, it was not feasible to conduct separate analyses for each gene. Instead, data from all sites were analyzed in a single combined matrix using a partitioned analysis under the GTR + I + Γ model (see above) with a molecular clock using MrBayes. Sites were partitioned into three categories based on reading frame (first, second, and third positions). Based on pilot runs, this by-codon-position partitioning scheme was strongly preferred over partitioning by gene identity (modal and mean log likelihood values sampled during MCMC runs were approximately 1657-fold higher in the by-codon partitioning scheme despite the fact that fewer free parameters were included). Five independent MCMC runs were performed for 1,034,000 generations (at which point the maximum standard deviation of split frequencies between runs was reported to be <0.03). Branch lengths were constrained to be clock-like, and a coalescent prior was used on branch lengths. Trees were sampled every 500 generations, and the first half of the samples was discarded as “burn-in,” resulting in a total of 5170 sampled trees.

As branch-length estimates obtained for a summary of clades (such as a majority-rule consensus tree) may be biased by the presence of polytomies, we assessed divergence of the RAVN clade from the other taxa on each of the sampled trees. Analyses were conducted without an outgroup because of the difficulty of obtaining reliable alignments between Marburg viruses and the sister lineage (Ebola virus). By enforcing the molecular clock, the tree was effectively rooted along the branch between the RAVN clade and the other sequences. This rooting position was strongly supported as the root position from the MrBayes analyses (all post-burn-in, sampled trees had this rooting position). This root position is also that which is obtained by midpoint rooting when a maximum likelihood tree is inferred, but without the molecular clock enforced (using PAUP* 4.0b10; Swofford 2002).

The test statistic we used to assess divergence of the RAVN clade was the proportion of total tree length contributed by the two branches that span the root of the tree. To be conservative, we assessed the smallest value of this test statistic of the 5170 trees from the MCMC sample, which was 0.5355 (the mean value over all sampled trees was 0.5763, and the median was 0.5764). We conducted Monte Carlo simulations to assess the probability that one would see a value of the test statistic that is this large under the null hypothesis that all of the Marburg virus sequences are drawn from a single, demographically exchangeable species. Trees were generated under the coalescent process using MCcoal (Rannala and Yang 2003) with a coalescent prior. For each simulated tree, the proportion of the total tree length contributed by the oldest branches in the tree (the two branches descending from the root of the tree) was calculated. If <5% of simulated trees showed a value of the test statistic more extreme (i.e., higher) than the real data set, then the divergence observed in the real sequences would not be compatible with the null hypothesis that all of the sequences were generated from a single demographically exchangeable population. As it remains unclear whether sequences from the 2005 Angola outbreak represent a single case of reservoir-to-human transfer or independent transmissions (Towner et al. 2006), analyses were also conducted on a pruned form of the matrix containing only one exemplar of the Angola outbreak.

To test for evidence that the samples of Marburg virus obtained from bats showed higher levels of sequence divergence compared with the sequences from humans, we performed a random pruning test. We measured the decrease in total tree length (sum of branch lengths) when we pruned the bat-derived sequences from the trees from the MCMC sample. Then, we performed a series of 1000 random deletions of the same number of taxa, and measured the reduction in tree length from these random prunings. If the bat-derived sequences resulted in a larger drop in tree length than 95% of these random prunings, then we would have some evidence of host-associated heterogeneity in the forces of molecular evolution acting on Marburg virus.

## Results

**Figure 1:**
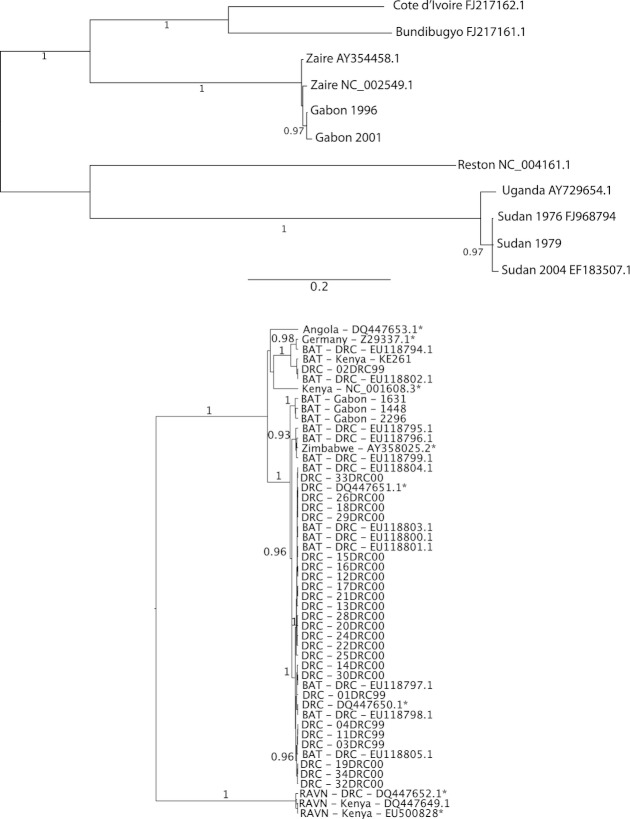
Phylogenetic trees estimated for Ebola and Marburg viruses, analyzed separately. (a) The majority-rule consensus tree of the Bayesian phylogenetic trees for the 11 Ebola sequences. Analyses were conducted without outgroups, so the tree should be interpreted as an unrooted network. Posterior probabilities from the combined analysis using the GTR + I + Γ models partitioned by gene are shown for internal branches that have posterior probabilities estimated to be >0.50. (b) The maximum a posteriori tree estimate of the genealogy of the Marburg sequences with maximum likelihood estimates of the branch lengths (under the GTR + I + Γ model with the molecular clock constraint). Posterior probabilities are shown for clades that have posterior probabilities estimated to be >0.50. Sequences are labeled by location and GenBank Accession number. See supplementary table (Tables S1 and S2) for a list of accession numbers for sequences that were created by concatenating multiple records. Bat-derived sequences are denoted by labels that start with “BAT.”

The basic phylogenetic results of our detailed analyses were clear and unambiguous, and did not conflict with the topologies presented in previous analyses (e.g., Towner et al. 2008). That is, among Ebola species, we see a basic tree topology that can be expressed as (Ebola Zaire + [Ebola Ivory Coast + Ebola Bundibugyo]) + [Ebola Sudan + Ebola Reston]), whereas among Marburg strains, the major feature of the trees centers on the split between “normal” strains and the two known examples of the RAVN strain. Figure 1a presents the majority-rule consensus tree for Ebola sequences from analysis of all genes partitioned by codon position. The individual gene analyses did not return any strongly supported relationships that conflicted with this tree. For example, analysis of 802 nucleotides of the VP30 gene placed Ebola Sudan with Ebola Ivory Coast + Ebola Bundibugyo, rather than with Ebola Reston, and analyses of the glycoprotein and L genes showed some conflict in the topology within the Ebola Zaire clade; in neither case were the conflicting topologies well supported. These examples of disagreement between trees estimated may be explained by random error from short sequence lengths or homoplasy, or could be evidence of recombination leading to different genealogies for different genes.

The Marburg RAVN strain is of particular interest in this set of analyses (Fig. 1b). Overall, sequence differentiation between RAVN and “normal” Marburg viruses is 21.0–21.4% (uncorrected *p*-distance across the entire genome), as compared with 31.6–40.4% among Ebola species. Past authors who have considered this strain and its taxonomic status have concluded that its differentiation does not merit species status (Sanchez et al. 1998). However, with this conclusion, RAVN is accorded a status equal to that of minor strains, such as strains Ratayczak, Popp, and Voege, all of which represent minor variants on the Marburg virus that was imported into Europe in 1967 (Fauquet et al. 2005); the biological importance of RAVN is thus lost.

**Figure 2:**
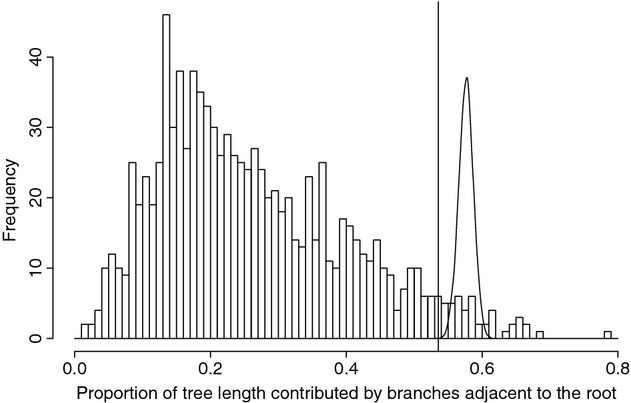
The null distribution of the divergence test statistic used to demonstrate that the RAVN sequences are more divergent than would be expected if all sequences were from a single species. The test statistic is the proportion of the tree length that is contributed by the two branches that are descendants of the root. The null distribution was generated by Monte Carlo simulations of coalescent genealogies followed by simulation of sequence data on those trees. The minimum value of the test statistic (0.536) observed in any of the sampled trees from the MCMC inference is shown as a line. The posterior distribution of the test statistic from the MCMC inference is shown as a continuous probability density (created using kernel density smoothing in R).

Our coalescent-based simulations, however, paint a different picture. More than 53.6% of the total tree length was contributed by the two branches separating the RAVN strains from the other Marburg virus sequences. Under the neutral coalescent, it is common for the oldest branches in the tree to be among the longest branches on the tree (Kingman 1982). However, the split between the two oldest groups in a coalescent tree is rarely as pronounced as that in the Marburg viruses. When we simulated sequences from a single species under the neutral coalescent, only 35 of the 1000 simulation replicates had values of the test statistic as large as this value (see Fig. 2), so the null hypothesis that the all of the strains could be drawn from a single unstructured population is rejected at *P* < 0.05. If we include only a single sample from the Angola outbreak, we must simulate smaller trees; here, still, 47 of 1000 replicates were more extreme than a basal branch length of 53.6%. If we use the posterior mean of the test statistic, rather than the smallest value observed in any sampled tree, we reject the single population hypothesis more firmly (only 18 of 1000 replicates in the null distribution with more extreme values, if we use all sequences from Angola; only 24 of 1000, if we consider the Angolan outbreak as the result of a single transmission to humans). Hence, the basal split on the Marburg virus tree, corresponding to the differentiation of the RAVN strain, was unexpectedly deep for these strains to have been sampled from a single evolving lineage.

Bat-derived filovirus genetic material, in contrast, was not markedly different from known human-derived strains. That is, sequences obtained from virus found in bats were scattered throughout the “normal” Marburg clade. Furthermore, bat-associated sequences did not have branch lengths longer other viral isolates (Fig. 1b). To assess this point, we pruned the 15 bat-derived sequences from the trees sampled during the MCMC run. This pruning resulted in an average reduction of total tree length of 0.0343 (branch lengths measured in expected changes per site). For comparison, we examined the effects of pruning 15 randomly chosen sequences from the “normal” Marburg sequences: 595 of 1000 random prunings produced more dramatic reductions in tree length, so lengths of branches in the tree provide no evidence that the bat-derived sequences are more divergent from samples obtained from humans. As shown in Fig. 1b, all “normal” Marburg sequences fall into one of seven subtrees defined by branches with strong support (posterior probability >0.95) and posterior mean branch lengths longer than 0.004. Four of these seven groups contain sequences derived from both humans and bats; these groups correspond to the outbreaks in 1999–2000 in the DRC, 1975 in Zimbabwe and South Africa, and 1967 in Germany. Only the sequences from the Musoke (Kenya 1980) and Angola (2005) human outbreaks are not closely related to known bat-derived sequences. The other distinct group corresponds to three sequences obtained from *Rousettus aegyptiacus* collected in Gabon (Towner et al. 2007); this clade has not been reported from human infections.

## Discussion

### Filovirus taxonomy

A recent review of basic tenets of virus taxonomy (Büchen-Osmond 2003) cited seven criteria by which viral species are differentiated. Relatedness of genome sequence and natural host range are the first two on this list. At first glance, it would be easy once again to dismiss the RAVN isolates as “not different enough” to merit recognition as a full species; it is true that RAVN and “normal” Marburg viruses are not as massively distinct from one another as the Ebola virus species are. However, without some way of distinguishing RAVN as particularly distinct, it has gotten lost and will continue to get lost “in the crowd” of minor variants—rather, RAVN represents a fascinating, unique lineage that may prove quite instructive regarding filovirus natural history and geography.

The phylogenetic simulations developed herein, however, paint a picture of impressive distinctiveness. The deep genomic differentiation *per se* present within Marburg virus is not the issue—were many deeply differentiated virus isolates to be known, producing a deeply divided, long-branched tree, the picture would be very different. Rather, in the present case, one long branch is present, and the diversity among isolates within the two terminal clades is low (Towner et al. 2006). This picture of minimal within-clade diversity, compared with massive among-clade differentiation, is precisely what suggests strongly that recognition as independent biologic entities (i.e., species) is warranted. Indeed, it is this phylogenetic structure that leads to the significant result in our simulations, particularly in the light of sympatric occurrence of the two viruses. Finally, we emphasize that the “minor” within-clade differentiation that characterizes non-RAVN Marburg viruses stretches the length of East Africa and southern Africa, showing only minimal differentiation, in sharp contrast to the dramatic differentiation of RAVN.

We emphasize that species status per se for RAVN is not our interest (although our opinion is that RAVN merits such status), but rather that it not “get lost” among other minor variants of Marburg and Ebola—rather, RAVN is quite distinct and merits careful consideration as to the biogeography and host relationships that can produce such a distinct lineage. If the taxonomic route were to be followed, considering the current taxonomic arrangement in the family (Netesov et al. 2002; Fauquet et al. 2005), we would recommend that the *Lake Victoria marburgvirus* strain Marburg RAVN should be removed from within *Lake Victoria marburgvirus* and recognized as a separate species, for which the name *Ravn marburgvirus* would be most logical. The type strain was derived from a case of Marburg hemorrhagic fever in Kenya in 1987 (Johnson et al. 1996), and the species is now also known from nearby areas of the DRC (Bausch et al. 2006), in both cases from sites where *Lake Victoria marburgvirus* is also known to occur. This change would leave *Marburgvirus* with two sympatric species, whereas *Ebolavirus* has five allopatric or parapatric species; once again, however, taxonomic recognition is not so critical, so long as RAVN is not forgotten among minor variants.

### Bat filovirus detections

Fifteen of the Marburg virus sequences analyzed herein were obtained from bats. We found no indication from the topology of the tree or the branch lengths that the bat-derived filoviruses represent a population distinct from viruses collected from human outbreaks. Bat sequences do not form a monophyletic group, nor are they associated with long or “deep” branches in the tree. This situation could indicate that the bats are the reservoir population, or that high gene flow exists between virus populations in bats and those in some other taxon that in truth acts as the reservoir; recently published information provides further support for these ideas (Towner et al. 2009). Given the short sequence lengths obtained from bat filovirus (about 302 nucleotides of the VP35 gene for most samples), it is also possible that we lack the power to detect subtle evidence for population structuring. Thus, firm conclusions about gene flow between the “bat” filovirus populations and the true source of human infections will require more data.

## Conclusions

**Figure 3:**
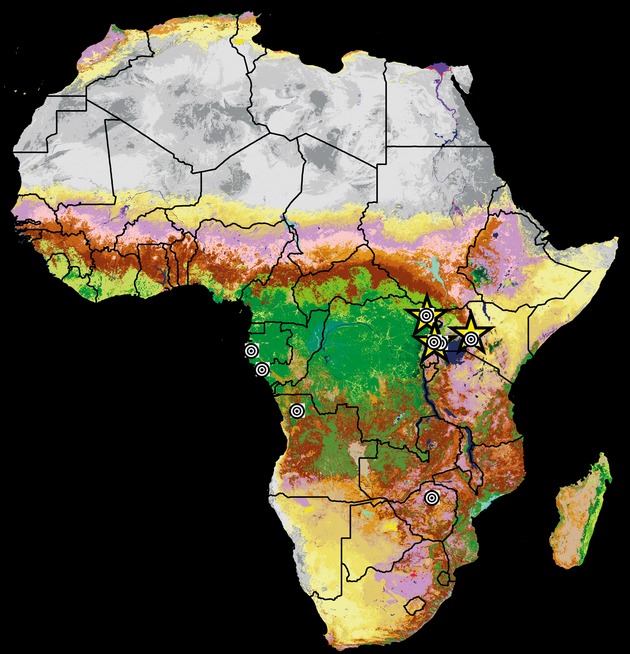
Map of known occurrences of Marburg virus strains across Africa. For reference, occurrences are shown on a background of land cover in 2000 (Mayaux et al. 2003). Occurrences of “normal” Marburg virus are shown as bulls eyes, whereas occurrences of RAVN are shown as yellow stars.

The previous picture of filovirus biogeography was one of a diverse Ebola virus, as sister to a monospecific Marburg virus (Peterson et al. 2004b, 2007). The coalescent-based simulation tests developed herein, however, suggest a different story: not only does the genus *Marburgvirus* appear to hold two distinct lineages, but two lineages that are broadly sympatric (Fig. 3). Indeed, the known range of RAVN is expanding as more studies are conducted (Towner et al. 2009), and “normal” Marburg has been isolated from all sites from which RAVN is known (Mt. Elgon, Kenya; Durba, DRC; Kitaka Cave, Uganda). The contrasting picture between Ebola and Marburg viruses thus morphs into a different sort of contrast—allospecies in Ebolavirus *versus* sympatric distinct lineages in Marburgvirus—such differences in phylogenetic community structure will eventually offer important insights into virus–host associations in this group (Vamosi et al. 2009).

These contrasts suggest key questions regarding filovirus natural history. Particularly, as the first steps are being taken toward definitive identification of the reservoir host of these viruses (Leroy et al. 2005; Swanepoel et al. 2007; Towner et al. 2007, 2009), and fuller appreciation of filovirus diversity is emerging (Negredo et al. 2011), consideration of these factors becomes important. For example, the fruit bat *Rousettus aegyptiacus* is now seen as central in the picture of Marburg virus maintenance: how then are two lineages of Marburg virus maintained in the same cave or mine? Recent work with bat populations in Uganda has even recovered both “normal” Marburg and RAVN from the same bat species (Towner et al. 2009), so the means of co-occurrence of the two remain unclear. In sum, more questions emerge as understanding increases; however, this study serves to clarify a key detail—Marburg virus is not monotypic, and the RAVN Marburg lineage should be considered carefully in studies of filovirus biogeography and evolution, as the lessons it has to offer will likely be quite informative.

## Supporting Information

**Table S1.** Key to Ebola sequence labels for concatenated sequences.

**Table S2.** Key to Marburg sequence labels for concatenated sequences.
