# Supplementary material for: Phylogenetic assessment of filoviruses: how many lineages of Marburg virus?
Source: Ecol Evol. 2012 Jul 1;2(8):1826–33. doi: 10.1002/ece3.297 (PMC3433987; doi:10.1002/ece3.297)
Supplement: Supplementary file 1 [file ece30002-1826-SD1.doc]

**Supplementary Tables:**

Table 1: Key to Ebola sequence labels for concatenated sequences

| **Label** | **GenBank Accession Numbers** |
| --- | --- |
| Gabon 1996 | AY058895.1, AY058898.1 |
| Gabon 2001 | AY526105.1, DQ205416.1 |
| Sudan 1976 | AF173836.1, U28134.1 |
| Sudan 1979 | U23069.1, U23458.1 |

Table 2: Key to Marburg sequence labels for concatenated sequences

| **Label** | **GenBank Accession Numbers** |
| --- | --- |
| BAT – Gabon - 2296 | EU068110.1, EU068113.1 |
| BAT – Gabon – 1448 | EU068108.1, EU068111.1 |
| BAT – Gabon – 1631 | EU068109.1, EU068112.1 |
| DRC – 01DRC99 | DQ466174.1, DQ466112.1, DQ466186.1, DQ466144.1 |
| DRC – 02DRC99 | DQ466184.1, DQ466116.1, DQ466189.1, DQ466145.1 |
| DRC – 03DRC99 | DQ466175.1, DQ466110.1, DQ466187.1, DQ466147.1 |
| DRC – 04DRC99 | DQ466185.1, DQ466111.1, DQ466190.1 |
| DRC – 05DRC99 | DQ466180.1, DQ466108.1, DQ466191.1, DQ466148.1 |
| DRC – 05DRC99-2 | DQ466179.1, DQ466109.1, DQ466149.1 |
| DRC – 06DRC99 | DQ466178.1, DQ466114.1, DQ466188.1, DQ466150.1 |
| DRC – 06DRC99 | DQ466177.1, DQ466115.1, DQ466151.1 |
| DRC – 07DRC99 | DQ466176.1, DQ466113.1, DQ466192.1, DQ466152.1 |
| DRC – 08DRC99 | DQ466183.1, DQ466118.1, DQ466195.1, DQ466153.1 |
| DRC – 10DRC99 | DQ466182.1, DQ466119.1, DQ466194.1, DQ466154.1 |
| DRC – 11DRC99 | DQ466181.1, DQ466117.1, DQ466193.1, DQ466155.1, |
| DRC – 12DRC00 | DQ466120.1, DQ466156.1 |
| DRC – 13DRC00 | DQ466122.1 |
| DRC – 14DRC00 | DQ466125.1, DQ466160.1 |
| DRC – 15DRC00 | DQ466121.1, DQ466157.1 |
| DRC – 16DRC00 | DQ466123.1, DQ466158.1 |
| DRC – 17DRC00 | DQ466124.1, DQ466159.1 |
| DRC – 18DRC00 | DQ466126.1 |
| DRC – 19DRC00 | DQ466127.1, DQ466169.1 |
| DRC – 20DRC00 | DQ466128.1 |
| DRC – 21DRC00 | DQ466129.1, DQ466170.1 |
| DRC – 22DRC00 | DQ466130.1 |
| DRC – 23DRC00 | DQ466132.1, DQ466162.1 |
| DRC – 24DRC00 | DQ466131.1, DQ466161.1 |
| DRC – 25DRC00 | DQ466143.1, DQ466171.1 |
| DRC – 26DRC00 | DQ466133.1, DQ466163.1 |
| DRC – 27DRC00 | DQ466172.1, DQ466134.1 |
| DRC – 28DRC00 | DQ466135.1 |
| DRC – 29DRC00 | DQ466136.1, DQ466164.1 |
| DRC – 30DRC00 | DQ466137.1, DQ466173.1 |
| DRC – 30DRC00-2 | DQ466140.1 |
| DRC – 31DRC00 | DQ466138.1, DQ466165.1 |
| DRC – 32DRC00 | DQ466139.1, DQ466166.1 |
| DRC – 33DRC00 | DQ466141.1, DQ466167.1 |
| DRC – 34DRC00 | DQ466142.1, DQ466168.1 |
